# The benefits of early palliative care on psychological well-being, functional status, and health-related quality of life among cancer patients and their caregivers: a systematic review and meta-analysis

**DOI:** 10.1186/s12904-025-01737-y

**Published:** 2025-04-28

**Authors:** Hartiah Haroen, Sidik Maulana, Hasniatisari Harun, Ristina Mirwanti, Citra Windani Mambang Sari, Hesti Platini, Novita Intan Arovah, Padila Padila, Shakira Amirah, Jerico Franciscus Pardosi

**Affiliations:** 1 Department of Community Health Nursing, Faculty of Nursing, Universitas Padjadjaran, Jl. Ir. Soekarno KM. 21, Jatinangor, West Java Sumedang, 45363 Indonesia; 2 Postgraduate Program of Nursing, Faculty of Nursing, Universitas Padjadjaran, Sumedang, 45363 Indonesia; 3 Department of Medical-Surgical Nursing, Faculty of Nursing, Universitas Padjadjaran, Sumedang, 45363 Indonesia; 4 Department of Critical Care and Emergency Nursing, Faculty of Nursing, Universitas Padjadjaran, Sumedang, 45363 Indonesia; 5 Department of Sports Science, Faculty of Medicine, Universitas Negeri Yogyakarta, Sleman, 55281 Indonesia; 6 Nursing Study Program, Faculty of Health Science, Universitas Muhammadiyah Bengkulu, Bengkulu, 38119 Indonesia; 7 Clinical Clerkship Program, Faculty of Medicine, Central of Jakarta, Universitas Indonesia; Dr, Cipto Manungunkusumo National General Hospital, Central Jakarta, 1043 Indonesia; 8 School of Public Health and Social Work, Faculty of Health, Queensland University of Technology, Brisbane, QLD 4000 Australia

**Keywords:** Early palliative care, Family caregiver, Functional status, Psychological well-being, Quality of life

## Abstract

**Background:**

Patients with cancer and their caregivers experience significant psychological, physical, and emotional burdens throughout the disease trajectory which reduces their quality of life (QoL). Early palliative care (EPC) has been proposed as a strategy to alleviate physical, psychological and emotional burdens and improve health outcomes. While evidence generally supports the benefits of EPC, variations in reported outcomes highlight the need for a deeper understanding of its impact across different patient populations and healthcare settings.

**Objective:**

The primary aim of this study was to evaluate the pooled effects of EPC on psychological, functional status, and QoL outcomes in both cancer patients and their caregivers. The secondary aim was to evaluate the satisfaction of the patients and their family caregivers.

**Methods:**

A systematic review and meta-analysis were conducted following the preferred reporting item for systematic review and meta-analysis (PRISMA) guidelines. Four databases, PubMed, Scopus, EBSCOhost, and Cochrane, were searched up to January 2024. This study included randomized controlled trial (RCT) and pilot-RCT studies reporting psychological outcomes (anxiety, depression), functional status, QoL, and satisfaction in cancer patients and their caregivers. Subgroup analysis was performed to explore the short-term (< 24 weeks) versus long-term (≥ 24 weeks) effects of EPC. Mean differences (MD) and standard mean differences (SMD) were calculated using a fixed-effects model according to the Mantel–Haenszel model and a random-effects model according to the DerSimonian and Laird method.

**Results:**

A total of 24 studies met our inclusion criteria. For cancer patients, EPC significantly reduced anxiety (MD = -0.62, 95% CI: -1.02; -0.23, *p* = 0.002) and improved QoL (SMD 0.13, 95%CI: 0.06; 0.19, *p* = 0.0004). However, there was no significant reduction in depression (SMD -0.15, 95% CI: -0.36; 0.05, *p* = 0.14) and improvement in functional status (MD = 2.14, 95% CI: -0.78; 5.06,* p* = 0.15). Subgroup analysis revealed that long-term EPC significantly reduced anxiety and depression while improving QoL, but had no significant effects on functional status. For caregivers, EPC did not significantly impact either physical or mental QoL (Short form/SF-36 physical: MD = 0.81, 95% CI: -0.46; 2.09, *p* = 0.21; SF-36 Mental: MD = 0.53, 95% CI: -1.03; 2.08, *p* = 0.51). Moreover, satisfaction was more likely to be higher in patients and their caregivers who received EPC than in those who received usual care (MD 2.45, 95% CI: 0.90; 4.01, *p* = 0.002, MD 4.09, 95% CI: 0.60; 7.58, *p* = 0.02, respectively).

**Conclusion:**

EPC reduces long term psychological burden and improve QoL and care satisfaction experience among patients with cancer. Therefore, EPC should be more broadly introduced into cancer care earlier to address patient’s psychological burdens.

**Supplementary Information:**

The online version contains supplementary material available at 10.1186/s12904-025-01737-y.

## Introduction

The global incidence of cancer in 2020 was 20 million new cases and 9.7 million deaths, and incidence of cancer is predicted to reach 35 million cases by 2050 [1]. Individuals with advanced cancer and their caregivers face a significant burden, which affects their psychological well-being. Additionally, continuous systemic treatment causes psychological distress due to uncertainty, anxiety, and fear [2]. According to previous meta-analysis, it is estimated that 30.6% of patients with advanced cancers with existential distress and 73.0% of patients with death anxiety [3]. Moreover, family caregivers with advanced cancers more likely have around 8 times to have initial major depressive episodes and 3 times to have generalized anxiety disorder than individuals in the general population [4]. Prolonged living with advanced cancer and continuous systemic treatment cause psychological distress due uncertainty, anxiety, dread, hopelessness, loss, and concerns regarding loved ones and alterations in social life [5]. Therefore, the demand for palliative care to address these issues is increasing among patients and caregivers [6]. However, palliative care is typically limited to end-of-life care, resulting in a substantial gap in meeting the needs of patients with advanced disease as early as possible during the trajectory of care [7, 8].

Early integration of palliative care into the provision of treatment for cancer patients, known as early palliative care (EPC), suggests that palliative care should be given to patients with advanced cancer at an earlier stage, specifically within 8 weeks following diagnosis [9]. EPC consists of three modalities including advanced communication to identify patient priorities, care and treatment coordination toward symptom management and control, and comprehensive psychosocial care for both patient and their family [10]. In conventional cancer treatment, palliative care is typically introduced after disease-modifying treatment has been deemed ineffective, no further therapeutic options are available, or death is imminent [11–13]. Conversely, EPC commences at an earlier stage in the progression of the disease and is more proximate to the diagnosis of an incurable form of cancer [10]. EPC involves the integration of palliative care with a standard of care at an early stage of the disease for cancer patients and their caregivers [9, 14]. Fundamentally, EPC adopts a proactive approach and is typically offered to patients who do not yet experience severe symptoms or significant psychosocial challenges [10]. EPC primarily aims to establish realistic and achievable treatment goals while supporting patient decision-making through comprehensive information, as well as evaluating their values and preferences in advance care planning [15]. Nevertheless, there is an ongoing discussion regarding the optimal timing of EPC, and there is a dearth of empirical information to guide patients in tailoring this strategy to their specific circumstances to meet their expectations and improve their quality of life (QoL) [16].

The effectiveness of EPC in patients with cancer remains debated. Some studies suggest that EPC interventions, including coordinated specialist palliative care approaches, significantly improve the QoL of patients and caregivers [10, 17]. However, EPC did not necessarily reduce depression and hospital admissions, and QoL, even in the last month of life [10, 18, 19]. The hospital admission may impact psychological well-being, and QoL [20–22]. Hospital admissions and length of stay can indicate poor functional status in cancer patients, as declining physical ability is often associated with an increased need for inpatient care due to complications or clinical deterioration [23]. Therefore, an evaluation of the pooled effect of EPC, especially on functional status, psychological well-being, and QoL among people living with cancer using meta-analysis is needed.

Previous meta-analyses have demonstrated a significant enhancement in psychology and QoL outcomes following the receipt of EPC, however the findings vary and are quite unclear due to the small sample size [13, 14, 24]. Moreover, the previous meta-analysis did not address the long-term effect and did not include their family caregiver outcomes [13, 14, 24, 25]. In many cases, cancer patients seek guidance from family members who are deeply familiar with them and significantly affected by these decisions [26]. These family caregivers take on various roles in supporting cancer treatment decision-making [26]. Family caregivers are regarded as the cornerstones of palliative care because they perform practical duties, offer emotional support, alleviate pain and other symptoms, and communicate with health services to enhance the QoL of a loved one [27]. Caregivers of advanced cancer patients often face physical exhaustion and psychological distress due to the illness's complexity and demands. The unpredictable disease progression and patient pain further heighten anxiety and depression, significantly impacting caregivers'quality of life [28].

According to the background above, it is necessary to conduct a study to enhance the substantiation regarding the impact of integrating EPC in improving the functional status, psychology, and QoL outcomes of patients with cancer and their family caregivers. The QoL of patients and their family caregivers appears to be affected by their satisfaction with the QoL [28, 29]. This study primarily aims to investigate the effectiveness of EPC on psychological well-being, functional status, and health-related quality of life (HRQoL) among cancer patients and their families, with a secondary aim of examining its effect on care satisfaction.

## Methods

### Study design

This study was a systematic review and meta-analysis. This study followed the Preferred Systematic Review and Meta-analysis (PRISMA) [30]. It was prospectively registered in the Prospective Register of Systematic Reviews (PROSPERO) with number CRD42025633007.

### Eligibility criteria

This study used the PICOS (Population, Intervention, Comparison, Outcome, Study) framework to determine the inclusion and exclusion criteria. The inclusion criteria were set for research articles on EPC interventions for individuals living with cancer and their caregivers, with clearly defined outcomes, such as psychological burden, functional status, and QoL.

Specifically, studies were included if they involved:

- Population of adult cancer patients (aged 18 years and older) and their caregivers. The patients include cancer patients with any specific cancer types and stages.
- Interventions involving EPC, initiated at diagnosis or early in the treatment process. Early palliative care is defined as integrating palliative support with standard cancer treatment soon after a patient is diagnosed with cancer.
- Comparisons with the usual care or standard palliative care group. Standard care refers to usual oncology treatment without systematic palliative care integration. Palliative care is provided only if deemed necessary by healthcare providers rather than through a structured approach.
- Outcomes measuring any of the following: (1) psychological distress refers to a range of common psychological conditions, from mild subclinical symptoms to clinically diagnosed disorders such as anxiety and depression [31]. Anxiety and depression was measured using standardized questionnaire (2) Functional status, is define as an individual's capacity to carry out daily living activities, measured using standardized questionnaire (3) QoL, defined as a sense of well-being in multidimensional perspective, measured using standardized questionnaire (4) Satisfaction, is define as patients'perceptions and responses to various aspects of their healthcare experience [32], measured using standardized questionnaire.
- Study including randomized controlled trials (RCT) studies and pilot of RCTs.

Case reports, non-research letters, editorials, invited commentaries, reviews, abstract-only articles, and preprints were excluded in this study to ensure the robustness and reliability of the synthesized data. Moreover, studies were omitted if they reported solely palliative interventions initiated or focused exclusively on end-of-life care. No restrictions were placed on the language of the publication to encompass a broad range of global research.

### Search strategy and study selection

A literature was systematically searched using multiple databases, including PubMed, Scopus, EBSCOhost, and the Cochrane Central Register of Controlled Trials (CENTRAL), to identify studies relevant to EPC for cancer patients and their caregivers. These databases were chosen because they are major databases that provide access to medical literature. This search was conducted covering the period from database inception to January 03, 2024, by two independent researchers (SM and SA). The search terms utilized were a combination of MeSH terms and free text words to encompass a wide range of studies on the topic: (("Palliative Care"OR"Supportive Care") AND (“Early palliative” OR"Early Intervention"OR"Early Stage") AND ("Cancer Patients"OR"Oncology Patients") AND ("Caregivers"OR"Family Caregivers")). The details of the search strategy are provided in Additional File 1.

To ensure a thorough retrieval of relevant literature, the ‘related article features'were used, and reference lists of included studies were hand-searched for additional sources. Studies were also sought by examining conference abstracts and proceedings to capture the most recent findings not yet published in journals. Following the initial electronic search, duplicates were meticulously removed, and titles and abstracts were screened to identify studies that met the eligibility criteria. Full texts of potentially relevant articles were then assessed for final inclusion in the review, and any discrepancies were resolved through discussion or consultation with a third reviewer (HH).

### Data extraction

Data extraction from the included studies was performed independently by two authors (SM and SA) using a standardized form to ensure consistency and comprehensiveness. The form captured essential details, such as author(s), publication year, study design, location, sample characteristics, patient demographics (age, gender), and outcomes of interest, including psychological well-being, functional status, QoL, and satisfaction. Any discrepancies in data extraction, including missing or inconsistent data, were addressed through discussion and consensus between the authors. If consensus could not be reached, a third reviewer (HH) was consulted. In cases of missing data, we attempted to obtain the necessary information by consulting supplementary sources and also directly contacting the study authors. If the missing data remained unavailable, they were excluded from the analysis with justification. The Risk of Bias (RoB) tool was used to evaluate the quality of the included studies. This scale facilitated the assessment of each study's quality based on five domains: randomization process, deviations of interventions, outcome data, outcome measurement, and reported results. Each domain was assessed as"low risk of bias,""some concerns,"or"high risk of bias."For studies assessed as having a high risk of bias, they were included due to their relevance and contribution to the overall analysis. Any discrepancies in the quality assessment were resolved through discussion among the authors, ensuring a consistent and fair evaluation of all included studies.

### Statistical analysis

Statistical analysis was conducted using Review Manager (RevMan version 5.4, The Cochrane Collaboration, Copenhagen, Denmark). Mean Differences (MD) were used to pool studies that applied the same measurement scale, while Standardized Mean Differences (SMD) were applied when studies used different measurement tools to assess the same outcome. The effect were analyzed using a fixed-effect model, based on the Mantel–Haenszel method, when heterogeneity was low. In cases of substantial heterogeneity, a random-effects model, following the DerSimonian and Laird method.

Heterogeneity among studies was quantitatively assessed using the inconsistency index (*I*^2^), with *I*^2^ > 50% signaling significant heterogeneity [33]. A random-effects model was consistently applied to all analyses, recognizing the inherent differences in study populations, interventions, and outcomes. To further understand the effects of EPC and identify potential causes of heterogeneity, we conducted subgroup analyses by effect duration, defined as studies that were categorized based on the reported duration of the palliative care's effects: short-term (less than 6 months) versus long-term (6 months or more). This analysis aimed to differentiate the immediate benefits of EPC interventions from their sustained impact over time. However, we did not proceed with meta-regression to further explore potential sources of heterogeneity. Moreover, we did not assess publication bias using funnel plots or Egger’s test, as each outcome included fewer than 10 studies, which could lead to unreliable results.

## Results

### Study selection

**Fig. 1:**
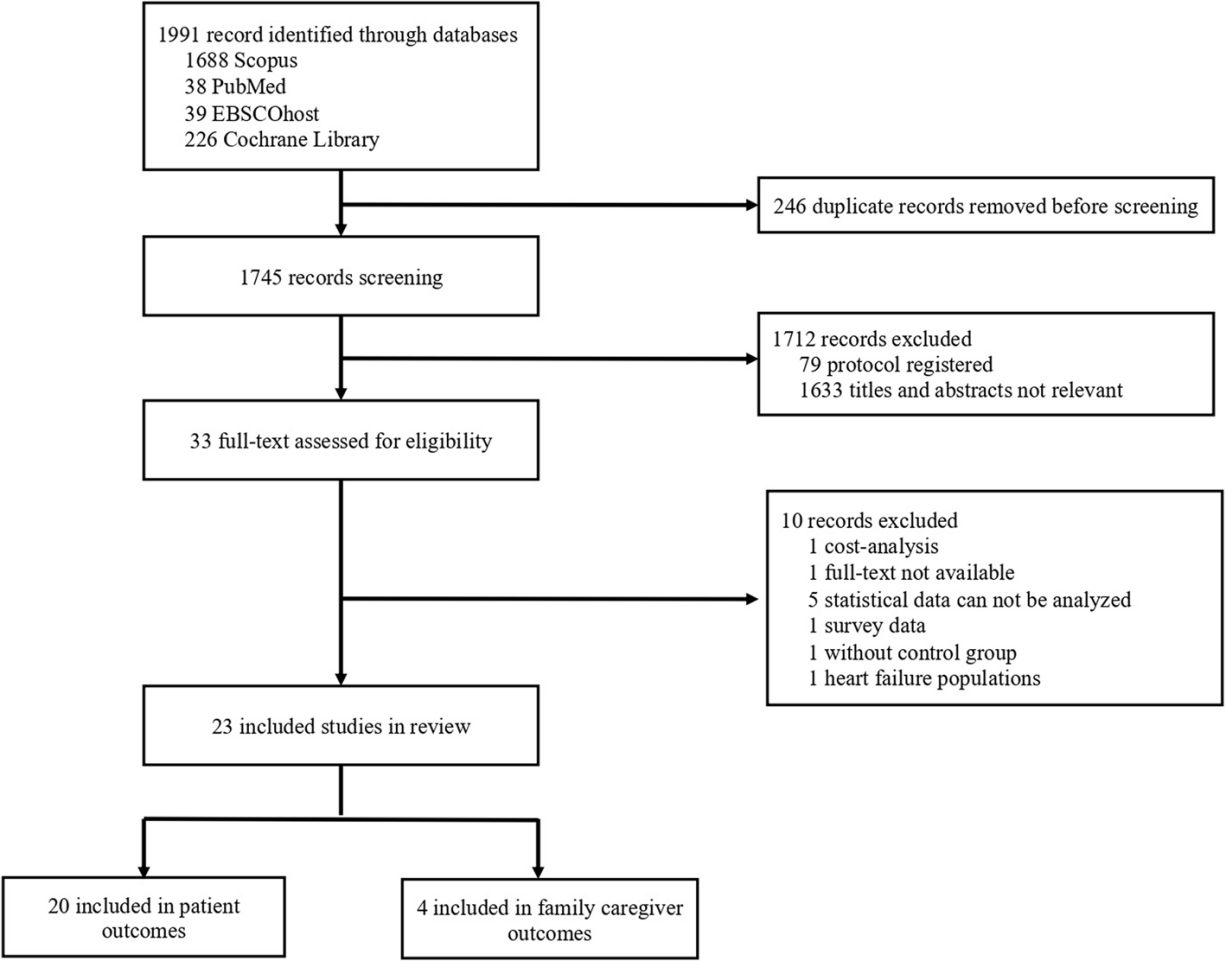
Study selection

A comprehensive search of the four databases yielded 1,991 records. After the removal of 246 duplicate entries, 1,745 records remained for screening. The titles and abstracts were reviewed, and 1,712 records were excluded. Subsequently, 33 full-text articles were assessed for their eligibility. Of these, ten were excluded because one was a cost analysis, one had no available full text, five contained statistical data that could not be analyzed, one was based on survey data, one lacked a control group, and one focused on populations with heart failure. Ultimately, 23 studies met the inclusion criteria for the systematic review [22, 34–55], with 20 addressing patient outcomes and 4 focusing on family caregiver outcomes (see Fig. 1).

### Characteristic of included studies

This study included 20 randomized controlled trials (RCTs) and 3 pilot RCTs conducted across multiple countries, including the United States, Canada, United Kingdom, Australia, China, Brazil, Italy, Denmark, Switzerland, Belgium, and the Czech Republic [22, 34–55]. The studies predominantly involved patients diagnosed with advanced-stage cancer, with sample sizes ranging from 30 to 468 participants. The study population included individuals with advanced solid tumors, lung cancer, gastrointestinal cancer, and other malignancies. Some studies have focused specifically on family caregivers of patients with cancer. The mean age of the patients varied across studies, with most participants being 60 years old. The proportion of male participants ranged from 21.9% to 82.8%, reflecting variability across different study populations.

The included studies also evaluated family caregivers of patients with advanced cancer, involving a total of 3 randomized controlled trials and 1 pilot RCT. The sample sizes ranged from 63 to 275 caregivers, with the proportion of male caregivers varying from 19.7% to 38.3%. The mean age of family caregivers spanned from 54.4 to 63.4 years. Caregivers were often spouses, children, or close relatives of patients diagnosed with lung cancer, gastrointestinal cancer, breast cancer, and other malignancies (See Table 1).

**Table 1:** Characteristics of included studies

Study	Location	Design	Population	Type of cancer	Male (%)	Age (Mean, SD)	Follow-up intervention	RoB
Bakitas et al. (2015) [34]	USA	RCT	207 patient with advanced-stage solid tumor	Lung (*n* = 88); GI Tract (*n* = 50); Breast (*n* = 23); Other solid tumor (*n* = 20); Genitourinary = 16); Hematological (*n* = 10)	IG: 53.85 CG: 51.46	IG: 64.03 (10.28) CG: 64.6 (9.59)	3 months	^a^
Brims et al. (2019) ​[35]​	UK and Australia	RCT	174 patient with malignant pleural mesothelioma	Malignant pleural mesothelioma (*n* = 174)	IG: 77.0 CG: 82.8	IG: 72.1 (66.7–77.7) CG: 72.8 (69.0–78.9)	12–24 weeks	^a^
Chen et al. (2022) [36]	China	RCT	120 patients with newly diagnosed NSCLC	Non-Small-Cell Lung Cancer (*n* = 120)	IG: 66.67 CG: 68.33	IG: 61.42 (11.48) CG: 64.62 (10.50)	24 weeks	^b^
Dionne-Odom et al. (2015) [37]	USA	RCT	124 family caregivers of advanced cancer patients	Lung (*n* = 53); GI Tract (*n* = 31); Breast (*n* = 10); Genitourinary (*n* = 10); Other solid tumor (*n* = 11); Hematological (*n* = 7)	IG: 23 CG: 19.7	IG: 61 (11.6) CG: 57.9 (11.9)	3 months	^a^
Dionne-Odom et al. (2022) [38]	USA	Pilot RCT	63 family caregivers of advance cancer patients	Breast (*n* = 9); Colon/rectal (*n* = 8); Lung (*n* = 6); Prostate (*n* = 5); Pancreatic (*n* = 2); Head and Neck (n = 3); Bladder/Kidney (*n* = 2); Other (*n* = 5)	IG: 21.9 CG: 22.6	IG: 63.4 (13.8) CG: 54.4 (13,5)	8–24 weeks	-
do Carmo et al. (2017) [39]	Brazil	Pilot RCT	63 patients with advanced cancer	Breast (*n* = 18); Colon and rectum (*n* = 7); Lung (*n* = 7); Head and neck (*n* = 4); Cervix (*n* = 9); Stomach (*n* = 6); Other (*n* = 10)	IG: 68.4 CG: 63.6	IG: 49.1 (11.1) CG: 57.0 (11.8)	90–180 days	^a^
El-Jawahri et al. (2017) [40]	USA	RCT	275 family caregiver of lung and gastrointestinal cancers	NA	IG: 31.4 CG: 30.4	IG: 57.5 (14.7) CG: 57.2 (12.5)	12 weeks	^b^
Eychmüller et al. (2021) [41]	Switzerland	RCT	150 patients with advanced cancer	Lung cancer (*n* = 55); Colorectal cancer (*n* = 17); Prostate cancer (*n* = 22); Breast cancer (*n* = 16); Urothelial cancer (*n* = 5); Pancreatic cancer (*n* = 35)	64.7	IG: 67.3 (59.0—74.7) CG: 67.3 (58.0—74.9)	2 months	^a^
Franciosi et al. (2019) [42]	Italy	RCT	281 patients with advanced cancer	Lung cancer (*n* = 163); Pancreatic (*n* = 60); Gastric (*n* = 44); Biliary (*n* = 14)	IG: 68 CG: 62	IG: 68.5 (11) CG: 68 (11)	12 weeks	^a^
Groenvold et al. (2017) [43]	Denmark	RCT	145 patients with advanced cancer	Lung cancer (*n* = 103); Digestive system (*n* = 58); Breast (n = 66); Other (*n* = 70)	IG: 43 CG: 41	NA	3–8 weeks	^a^
Johnsen et al. (2020) [44]	Denmark	RCT	297 patients with advanced cancer	Lung cancer (*n* = 103); Digestive system (*n* = 58); Breast (*n* = 66); Other (*n* = 70)	IG: 43 CG: 41	NA	8 weeks	^a^
McDonald et al. (2017) [22]	Canada	RCT	182 primary caregivers	Lung cancer (*n* = 30); Gastrointestinal (*n* = 67); Genitourinary (*n* = 32); Breast (*n* = 32); Gynaecological (*n* = 21)	IG: 38.3 CG: 30.7	IG: 58.0 (25–83) CG: 58 (61.7)	4 months	^a^
Maltoni et al. (2016) [45]	Italy	RCT	207 patients with advanced pancreatic cancer	Pancreatic cancer (*n* = 207)	IG: 61.5 CG: 52.8	IG: 67 (43–85) CG: 66 (31–84)	12 weeks	^c^
McCorkle et al. (2015) [46]	USA	RCT	146 patients with late-stage cancer	NR	43.8	60 (27–87)	3 months	^c^
Rodin et al. (2022) [47]	Canada	RCT	232 patients with advanced cancer	Breast cancer (*n* = 72); Gastrointestinal (*n* = 139); Genitourinary (*n* = 78); Gynecological (*n* = 71); lung (*n* = 101)	IG: 50.5 CG: 52.7	IG: 61.6 (11.8) CG: 61.4 (12.1)	4 months	^b^
Scarpi et al. (2018) [48]	Italy	RCT	186 patients with advanced gastric cancer	Gastric cancer (*n* = 186)	68.2	IG: 70 (36–84) CG: 69 (34–89)	12 weeks	^c^
Schenker et al. (2018) [49]	USA	Pilot RCT	30 patients with pancreatic cancer	Pancreatic cancer (n = 30)	50	63 (11)	3 months	^b^
Slama et al. (2020) [50]	Czech Republic	RCT	126 patients with advanced cancer	Head and neck (n = 3); esophagus and stomach (n = 34); lungs (n = 26); pancreas (n = 37); colon (n = 26)	59.5	IG: 61.1 (9.8) CG: 63.5 (10.4)	3–6 months	^b^
Tamel et al. (2010) [51]	USA	RCT	151 patients with NSCLC	Non-small-cell lung cancer (*n* = 121)	51.6	IG: 64.98 (9.73) CG: 64.87 (9.41)	12 weeks	^a^
Temel et al. (2017) [52]	USA	RCT	350 patients with Lung and GI cancer	Lung cancer (*n* = 191); GI cancer (*n* = 159)	54	IG: 65.64 (11.26) CG: 64.03 (10.46)	12–24 weeks	^a^
Temel et al. (2020) [53]	USA	RCT	405 patients with advanced lung and GI cancer	Lung cancer (*n* = 234); Esophageal/gastroesophageal (*n* = 33)	56.5	IG: 65.5 (9.4) CG: 65.0 (10.7)	12–24 weeks	^a^
Vanbutsele et al. (2018) [54]	Belgium	RCT	468 patients with advanced cancer	Gastrointestinal (*n* = 71); lung (*n* = 51); head and neck (*n* = 19); breast (*n* = 14); melanoma (*n* = 15); genitourinary (*n* = 16)	68.8	IG: 64.5 (57.3–71.0) CG: 65.0 (57.0–71.0)	12 weeks	^a^
Zimmermann et al. (2014) [55]	Canada	RCT	461 patients with advanced cancer	Lung (*n* = 101); gastrointestinal (*n* = 139); genitourinary (*n* = 78); breast (*n* = 72); gynaecological (*n* = 71)	57,8%	IG: 61.2 (12.0) CG: 60.2 (11.3)	1–4 months	^b^

*RCT* randomized controlled trial, *IG* intervention group, *CG* control group, *RoB* risk of bias

^a^Low, ^b^some concern, ^c^high

The interventions included in these studies involved EPC, with varied qualifications for facilitators. For instance, some interventions were delivered by specialized palliative care teams, consisting of physicians and advanced practice nurses, while others utilized trained lay navigators or nurses. The interventions often included structured, multidisciplinary care, with a focus on symptom management, psychosocial support, and decision-making, starting at different points after diagnosis, ranging from within weeks to a few months. The details of these interventions can be found in Table 2.

**Table 2:** Characteristics of intervention, measurement tool, and summarize of results

**Study**	**Start time of intervention**	**Intervention**	**Control**	**Measurement tools**	**Results**^**a**^
Bakitas et al. (2015) [34]	30 to 60 days after diagnosis of advanced cancer	Standard of oncology care and six structured weekly telephone coaching sessions conducted by an advanced practice nurse	Standard of oncology care and access to PC team only if requested	FACIT-PAL	*p* = 0.34
Brims et al. (2019) [35]​	3 weeks of randomization	Specialist palliative care (SPC) visit every 4 weeks witch include SPC consultation and additional treatment and or referrals as needed	Standard of oncology care and referral to SPC based on clinical need	SF- 36 physic SF- 36 mental FAMCARE	*p* = 0,37 *p* = 0.63 *p* = 0.003
Chen et al. (2022) [36]	8 weeks after diagnosis	Patients meet the palliative care team. The major of intervention include pain, nutrition, psychology, and QoL	Referral to palliative care based if requested	HADS-A HADS-D FACT-G	*p* = 0.136 *p* < 0.001* *p* < 0.017*
Dionne-Odom et al. (2015) [37]	30 to 60 days after diagnosis of advanced cancer	Standard of oncology care and six structured weekly telephone coaching sessions conducted by an advanced practice nurse	Standard of oncology care and access to PC team only if requested	CQOL-C	*p* = 0.37
Dionne-Odom et al. (2022) [38]	within 60 days after diagnosis of advanced cancer	Patients received six weekly, one-on-one phone coaching sessions (20–60 min each) led by specially trained palliative care lay navigators, covering topics such as stress management, seeking help, organizing health information, self-care, and decision-making in serious illness	NA	HADS-A HADS-D FACIT-PAL CQOL-C	Patient (cohen d) HADS-A (0.17) HADS-D (− 0.39) FACIT-PAL (− 0.27) Family caregiver HADS-A (− 0.44) HADS-D (− 0.1) CQOL-C (− 0.07)
do Carmo et al. (2017) [39]	2–3 weeks of randomization	Patients received five weekly individual psychosocial sessions using CBT techniques, including psychoeducation about their clinical condition, anxiety management, and cognitive restructuring. Early PC appointments with PC physicians were scheduled after the first two psychosocial sessions	Participants received early PC intervention alone, with PC physician visits scheduled every 3 ± 1 weeks	HADS-A HADS-D PHQ- 9	*p* < 0.001* *p* = 0.017* *p* = 0.029*
El-Jawahri et al. (2017) [40]	4 weeks of randomization	Patients met with a board-certified palliative care physician or advanced-practice nurse, with additional visits scheduled as needed by the patient, caregiver, oncologist, or PC clinician. The intervention focused on symptom management, coping strategies, illness understanding, treatment decisions, advance care planning, and disposition discussions	Referral to palliative care based if requested	SF- 36 physic SF- 36 mental	*p* = 0.669 *p* = 0.679
Eychmüller et al. (2021) [41]	8 weeks after diagnosis of incurable cancer and 16 weeks of randomization	Patients received usual oncology care plus a single structured 50-min intervention conducted by a trained physician and nurse using the SENS framework (Symptoms, End-of-Life Decisions, Network, and Support for Carers). They were encouraged to bring a family member to the session	Referral to palliative care based if requested	FACT-G	*p* = 0.62
Franciosi et al. (2019) [42]	8 weeks after diagnosis and 2 weeks after randomization	PC intervention provides a comprehensive, patient-centered approach by addressing physical symptoms, as well as social and spiritual needs. The PC intervention is delivered by board-certified oncologists and palliative care specialists, with full-time involvement of nurses, ensuring a multidisciplinary approach to care	Oncology treatment without structured palliative care integration	FACT-G	*p* < 0.001*
Groenvold et al. (2017) [43]	Earlier time than would otherwise have been the case	Intervention consisted of specialist palliative care initiated earlier than usual. Patients were referred to an SPC team, and the number, frequency, and type of SPC contacts were determined based on individual patient needs. The intervention was multidisciplinary and adapted to each patient, with no additional guidelines developed beyond existing SPC practices. No specific manual was used, and intervention fidelity was not formally assessed	No report	EORTC QLQ	*p* = 0.14
Johnsen et al. (2020) [44]	8 weeks of randomization	Intervention consisted of specialist palliative care initiated earlier than usual. Patients were referred to an specialized palliative care (SPC) team, and the number, frequency, and type of SPC contacts were determined based on individual patient needs. The intervention was multidisciplinary and adapted to each patient, with no additional guidelines developed beyond existing SPC practices	Patients received basic palliative care but were not referred to specialized palliative care teams	HADS-A HADS-D EORTC QLQ FAMCARE	*p* = 0.058 *p* = 0.328 *p* = 0.541 *p* = 0.210
McDonald et al. (2017) [22]	NA	Patients received early palliative care alongside standard oncology care, which included consultation and monthly follow-up in an outpatient palliative care clinic led by a palliative care physician and nurse. For patients, the intervention focused on symptom management, goal setting, advance care planning, and addressing social, emotional, and spiritual needs. Caregivers received social support, emotional care, and resource assistance to help in patient care. Additionally, a nurse conducted follow-up phone calls a week after each visit, and 24-h telephone support was available from palliative care physicians. Ancillary interventions were provided based on patient needs, including home nursing, transfer of care to a home palliative care physician, and access to an acute palliative care unit	Patients received standard oncology care, consisting of outpatient follow-up by an oncology physician and nurse, with visit frequency determined by chemotherapy and radiation schedules. There was no routine psychosocial assessment for patients or caregivers. However, patients could be referred to the palliative care team at any time based on perceived need	SF- 36 physic SF- 36 mental CQOL-C FAMCARE	*p* = 0.20 *p* = 0.60 *p* = 0.51 *p* = 0.02*
Maltoni et al. (2016) [45]	2 weeks of enrollment	Patients had a scheduled appointment with a palliative care specialist within 2 weeks of enrollment and continued follow-ups every 2–4 weeks until death. palliative care appointments were guided by general palliative care guidelines, and the full-time palliative care specialist could prescribe medications and request interventions addressing physical, psychological, and spiritual needs. However, any recommendations on decision-making had to be shared with the oncologist	Patients were not scheduled for palliative care consultations unless requested by the patient, family, or attending oncologist	FACT-Hep	*p* = *0.30*
McCorkle et al. (2015) [46]	Exact timing is not explicitly stated (probably around 100 days after diagnosis)	Patients received a 10-week standardized intervention delivered by a multidisciplinary team. Key components included monitoring patient status, symptom management, complex care procedures, patient and caregiver education, care coordination, and quality of life enhancement. Goals of care were also discussed. Patients were contacted by an Advanced Practice Nurse (APN) within 24 h of enrollment, followed by weekly phone and in-person contacts, consisting of five clinic visits and five telephone calls	Patients received routine oncology care but did not receive the APN-coordinated intervention	HADS-A PHQ- 9 FACT-G	*p* = 0.325 *p* = 0.969 *p* = 0.106
Rodin et al. (2022) [47]	1 month of recruitment	Intervention consisted of four main components. First, patients received a comprehensive, multidisciplinary, in-person assessment (60–90 min) within 1 month of recruitment, focusing on symptoms, psychological distress, social support, and home services. Second, a palliative care nurse conducted routine telephone follow-ups 1 week after the initial consultation and continued as needed. Third, patients had monthly outpatient palliative care follow-ups (20–50 min). Fourth, a 24-h on-call service was available for urgent issues. Additional interventions, such as home nursing, palliative physician care, or transfer to an inpatient palliative care unit, were provided based on the patient’s needs	No report	FAMCARE	*p* = 0.13
Scarpi et al. (2019) [48]	2 weeks of enrollment	Patients met a palliative care (PC) physician within 2 weeks of enrollment and had follow-ups every 2 to 4 weeks until death. Additional non-scheduled PC consultations were allowed based on clinical need, and researchers could use routine assessment tools not included in the study. PC interventions followed general PC guidelines and were managed solely by physicians with clinical experience in palliative care, though most lacked formal PC training. The full-time PC specialist could prescribe medications and request additional interventions for physical, psychological, and spiritual needs, but treatment decisions had to be shared with the oncologist	Patients were not scheduled for PC consultations unless requested by the patient, their family, or the attending oncologist	FACT-G	*p* = 0.167
Schenker et al. (2018) [49]	8 weeks after diagnosis of advanced metastatic	Patients received in-person palliative care visits with a specialty-trained palliative care physician, scheduled in the same building as oncology appointments. Follow-up visits occurred monthly for the first three months, with additional visits as needed. Patients did not incur costs for palliative care visits and received travel reimbursements ($40 for the first visit, $25 for later visits if not on the same day as an oncology appointment). Before each visit, patients completed surveys assessing symptom burden (Edmonton Symptom Assessment Scale [ESAS]) and distress (Distress Thermometer), while caregivers completed assessments for caregiver burden and distress. To facilitate communication, an email was sent to both the oncologist and palliative care physician before and after each visit to provide updates on patient symptoms, distress, and goals of care.	Standard of oncology care and access to PC team only if requested by their oncologist	HADS-A HADS-DPHQ	Patient HADS-A: CG > IG HADS-D: CG > IG PHQ: CG > IG Family caregiver HADS-A: IG > CG HADS_D: IG > CG
Slama et al. (2020) [50]	Six weeks after diagnosis of advanced cancer	Intervention consisted of consultations with a palliative care physician every 6 to 8 weeks. Each consultation included pain and symptom assessment using the Edmonton Symptom Assessment Scale (ESAS), evaluation of coping strategies and psychosocial support needs, and recommendations for pharmacological symptom management. The first visit lasted approximately 45 min, while follow-up visits lasted 20 min. In addition to symptom management, the palliative care specialist explored patients'psychological and spiritual needs, referring them to a social worker, psychologist, or chaplain if necessary	Patients received on-demand palliative care consultations only when requested by the treating oncologist	HADS-A HADS-D	p = 0.883 p = 0.835
Tamel et al. (2010) [51]	8 weeks after diagnosis	Patients received early palliative care integrated with standard oncologic care, beginning with a consultation with a palliative care team (board-certified palliative care physicians and advanced-practice nurses) within 3 weeks of enrollment, followed by at least monthly outpatient visits until death. Additional visits could be scheduled based on the patient’s, oncologist’s, or palliative care provider’s discretion. The intervention followed general guidelines from the National Consensus Project for Quality Palliative Care, focusing on physical and psychosocial symptom management, goal setting, treatment decision-making, and care coordination	Patients received standard oncologic care alone, with no scheduled palliative care visits unless specifically requested by the patient, family, or oncologist	FACT-L	*p* = 0.03*
Temel et al. (2017) [52]	8 weeks after diagnosis of incurable cancer and 4 weeks of enrollment	Patients with a member of the outpatient PC team within 4 weeks of enrollment and had at least one visit per month until death. The PC team, consisting of physicians and advanced practice nurses, followed the National Consensus Project for Quality Palliative Care guidelines. If an in-person visit was not possible, PC clinicians contacted patients via telephone, and additional visits could be scheduled at the discretion of the patient, oncologist, or PC clinician	Patients received standard of oncology care and patients could only meet with a PC clinician upon request by the oncologist, patient, or family	PHQ FACT-G	*p* = 0.991 *p* = 0.430
Temel et al. (2020) [53]	8 weeks after diagnosis of incurable cancer and 4 weeks of enrollment	Patientsmet with a PC physician or advanced practice nurse (APN) within four weeks of enrollment and had at least monthly visits until death, scheduled alongside oncology appointments. If an in-person visit was not possible, PC clinicians conducted telephone follow-ups. Additionally, a PC team member was required to visit hospitalized patients	Patients could only see a PC clinician upon request by the oncologist, patient, or caregiver, without a structured follow-up schedule	HADS-A HADS-D FACT-G	*p* = 0.06 *p* = 0.85 *p* = 0.19
Vanbutsele et al. (2018) [54]	12 weeks after diagnosis	Patients received structured monthly consultations with a specialized palliative care nurse starting within 3 weeks of enrollment and continuing until death. These consultations, based on a semi-structured interview form, focused on illness understanding, symptom burden, psychological and spiritual coping, and medical decision-making. Monthly symptom assessments using the Edmonton Symptom Assessment Scale (ESAS) were conducted, with nurses discussing significant symptom changes with the patient and oncologist. The intervention was nurse-led, unlike previous physician-led models, and aimed to integrate palliative care into oncology through the participation of palliative care nurses in weekly multidisciplinary oncology meetings and documentation in the electronic patient file	Patients had an introductory consultation with a specialist oncology nurse, dietician, and psychologist at the start of treatment, but follow-up consultations were based on patient preference. Palliative care involvement was not systematic and was only provided on demand, often late in the disease trajectory	EORTC QLQ	*p* = 0.12
Zimmermann et al. (2014) [55]	NA	Patients involved a multidisciplinary assessment within 1 month of recruitment (60–90 min), routine telephone follow-ups by a nurse, monthly outpatient visits (20–50 min), and 24/7 telephone support. Additional services included home nursing care, referrals to home palliative physicians, or admission to the palliative care unit when needed	Patients received standard oncology care with palliative referrals available upon request	FAMCARE	*p* < 0.0001*

*NA* not available, *DASS-A* Depression, Anxiety, Stress Scale – Anxiety, *DASS-D* Depression, Anxiety, Stress Scale – depression, *PHQ- 9* Patient Health Questionnaire- 9, *FACIT-PAL* functional Assessment of Chronic Illness Therapy – Palliative Care, *FACIT* The Functional Assessment of Cancer Therapy, *EORTC QLQ-C30* the European Organization for Research and Treatment of Cancer Quality of Life Questionnaire-C30, *SF- 36* Short Form Health Survey- 36 items, *CQOL-C* The Caregiver Quality of Life Index-Cancer, *FAMECARE P- 16* Patients completed a 16-item measure of patient satisfaction, *FAMECARE*- *2* Family Satisfaction with End-of-Life Care

^a^Value at the maximum follow-up measurement

*statistically significant

**Fig. 2:**
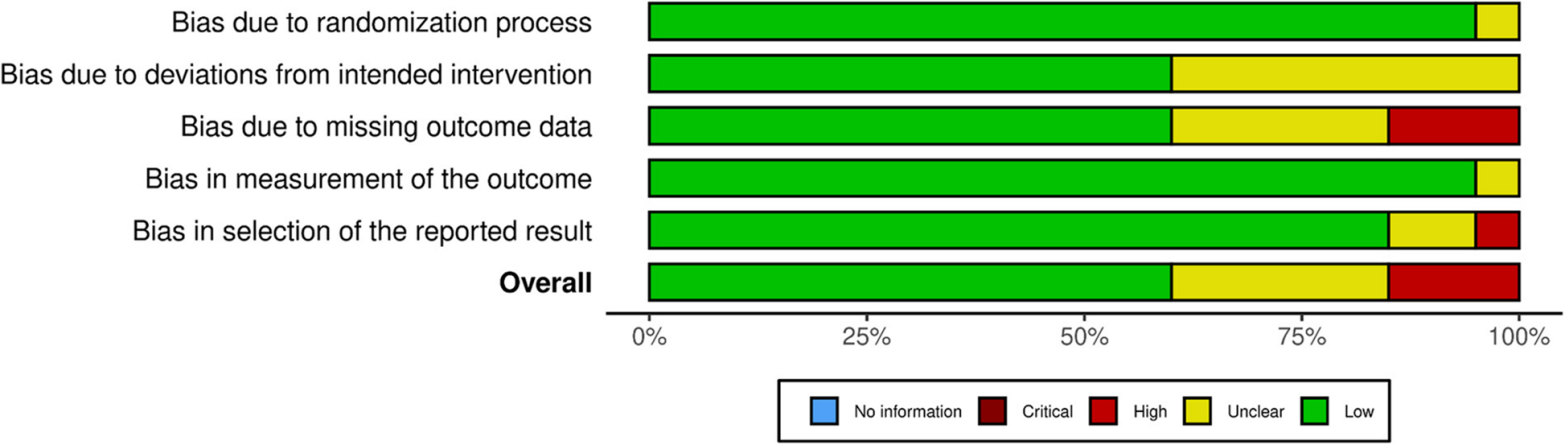
Summary risk of bias

Risk of bias assessments indicated that the majority of the studies had a low risk of bias, while some showed concerns or were rated as high risk (see Fig. 2 for summary risk of bias and Additional File 2 for risk of bias’ traffic light plot of individual studies). Specifically, studies such as Maltoni et al., McCorkle et al., and Scarpi et al. were identified as having a high risk of bias, primarily due to missing outcome data (D3) and concerns related to the selection of reported results (D5). These limitations may introduce potential biases in effect estimation and study interpretation.

### Effect estimates of EPC to patient and family caregiver outcomes

Anxiety and depression as measured by Depression, Anxiety, Stress Scale – Anxiety (DASS) in patient who received EPC was significantly low compared to patient in usual care with MD = − 0.62 (95% CI: − 1.02; − 0.23, *p* = 0.002) and MD − 1.40 (95% CI: − 2.40; − 0.39, *p* = 0.006), respectively (see Table 2 and Supplementary file 3). However, depression as measured using all of kind measurement (eg. DASS-D and Patient Health Questionnaire/PHQ) shows were not significant. The QoL of patients in the EPC group was significantly improved compared to usual care (SMD 0.13, 95%CI: 0.06; 0.19, *p* = 0.0004), particularly when QoL measured by The Functional Assessment of Cancer Therapy (FACT) with MD 2.36 (95%CI: 0.40; 4.32, *p* = 0.02). However, there was no significant difference in functional status between patients who received EPC and usual care with MD of Functional Assessment of Chronic Illness Therapy – Palliative Care (FACIT-PAL) 2.14 (95% CI: − 0.78; 5.60, *p* = 0.15). Additionally, depression as measured by the PHQ showed that patients who received EPC were significantly higher than those in the usual group (MD 0.76, 95% CI: 0.12; 1.39, *p* = 0.02).

For family caregivers, the meta-analysis showed no significant difference in QoL between groups. Neither the physical nor mental components of the SF- 36 scale showed significant improvements (Short Form Health Survey- 36 items/SF- 36 Physical: MD = 0.81, 95% CI: − 0.46; 2.09, *p* = 0.21; SF- 36 Mental: MD = 0.53, 95% CI: − 1.03; 2.08, *p* = 0.51, respectively). Additionally, the Caregiver Quality of Life Index-Cancer (CQOL-C) also did not show significant difference (MD = − 4.08, 95% CI: − 9.41, 1.25, *p* = 0.13, *I*^2^ = 0%).

The patients and family caregivers receiving EPC reported significantly higher satisfaction with care than those receiving usual care (MD = 2.45 (95% CI: 0.90; 4.01, *p* = 0.002) and 4.09 (95% CI: 0.60 to 7.58, *p* = 0.02), respectively. The effect estimate of EPC on patient and family caregivers can be seen in Table 3, and the forest plot can be seen in Additional File 3.

**Table 3:** Meta-analysis of pooled effect EPC to patient and family caregiver outcomes

Outcome	Number of unique studies^**^	MD	95%CI	*p*	*I*^*2*^	Reference
**Patient outcome**						
Anxiety (DASS-A)	10	− 0.62	− 1.02, − 0.23	0.002^*^	9%	[36, 38, 39, 44, 45, 49, 53]
Depression	15	SMD = − 0.15	− 0.36, 0.05	0.14	69%	[38, 39, 44, 46, 49, 52, 53]
DASS-D	9	− 1.40	− 2.40, − 0.39	0.006^*^	67%	[36, 38, 39, 44, 49, 53]
PHQ- 9	6	0.76	0.12, 1.39	0.02	38%	[39, 46, 49, 52]
FACIT-PAL	4	2.14	− 0.78, 5.06	0.15	0%	[34, 38]
Quality of life	17	SMD = 0.13	0.06, 0.19	0.0004^*^	47%	[41–46, 48, 50–54]
FACT	12	2.36	0.40, 4.32	0.02^*^	31.9%	[36, 41, 42, 48, 51–53]
EORTC QLQ-C30	6	2.27	− 0.58, 5.12	0.12	32%	[43, 44, 50, 54]
Satisfaction (FAMCARE-P- 16)	3	2.45	0.90, 4.01	0.002^*^	0%	[44, 47, 55]
**Family caregiver outcome**						
Quality of life	13	SMD = 0.05	− 0.05; 0.14	0.34	0%	[22, 35, 37, 38, 40]
SF- 36 Physic	5	0.81	− 0.46, 2.09	0.21	0%	[22, 35, 40]
SF- 36 Mental	5	0.53	− 1.03, 2.08	0.51	0%	[22, 35, 40]
CQOL-C	3	− 4.08	− 9.41, 1.25	0.13	0%	[22, 37, 38]
Satisfaction (FAMCARE- 2)	2	4.09	0.60, 7.58	0.02^*^	79%	[22, 35]

*MD* Mean difference, *SMD* Standard mean difference, *DASS* Depression, Anxiety, Stress Scale, *PHQ- 9* Patient Health Questionnaire- 9, *FACIT-PAL* Functional Assessment of Chronic Illness Therapy – Palliative Care, *FACT-G* Functional Assessment of Cancer Therapy – General, *EORTC QLQ-C30* the European Organization for Research and Treatment of Cancer Quality of Life Questionnaire-C30, *SF- 36* Short Form Health Survey, *CQOL-C* The Caregiver Quality of Life Index-Cancer, *FAMECARE P- 16* Patients completed a 16-item measure of patient satisfaction, *FAMECARE- 2* Family Satisfaction with End-of-Life Care

^*^Statistically significant

^**^Unique studies refer to the number of studies analyzed, which may include multiple studies derived from the same original study but with different measurements or assessments of both short-term and long-term effects

We further conducted a sensitivity analysis and found that the depression outcome study by Temel et al. (2017) was an outlier. When this study was excluded, heterogeneity decreased to 65%, with an SMD of − 0.20 (95% CI: − 0.41 to 0.01). In the QoL outcome, the study by Temel et al. (2017) was identified as an outlier. When excluded, heterogeneity dropped to 22%.

### Short and long-term effect of EPC for patient outcome

The meta-analysis also assessed short-term (< 24 weeks) and long-term (≥ 24 weeks) patient outcomes. In the short-term, anxiety, depression, functional status, and QoL were not significantly different (p ≥ 0.05). In the long term (≥ 24 weeks), anxiety and depression as measured by DASS in patients who received EPC were significantly lower compared to patients in usual care with MD = − 0.84 (95% CI: − 1.40; − 0.28, *p* = 0.003) and MD − 2.39 (95% CI: − 4.30; − 0.47, *p* = 0.01), respectively. However, depression as measured using all of the kind measurements showed was not significant. QoL was more likely to be higher than usual care (SMD 0.25, 95% CI 0.12, 037, *p* < 0.0001). Notably, long-term effects on functional status did not show significant differences between groups in long term effect (*p* ≥ 0.05) (See Table 4 and the forest plot in Additional File 3).

**Table 4:** Sub-group analysis of effect EPC to patient outcome, according measurement follow-up

Outcome	Number of studies**	MD	95%CI	*p*	*I*^*2*^	Reference
** < 24 weeks follow-up**						
Anxiety (DASS-A)	5	− 0.41	− 0.97, 0.15	0.15	0%	[38, 39, 44, 53]
Depression		− 0.03	− 0.21, 0.16	0.78	40%	[38, 39, 44, 46, 49, 52, 53]
DASS-D	5	− 0.55	− 1.40, 0.30	0.21	21%	[38, 39, 44, 49, 53]
PHQ- 9	4	0.52	− 0.31, 1.35	0.22	57%	[39, 46, 49, 52]
FACIT-PAL	2	2.39	− 1.22, 5.99	0.19	0%	[34, 38]
Quality of life	12	SMD = 0.07	− 0,02, 0.15	0.12	45%	[41–46, 48, 50–54]
FACT	7	1.53	0.65, 3.70	0.17	53%	[41, 42, 46, 48, 51–53]
EORTC QLQ-C30	4	1.54	− 1.61, 4.68	0.34	49%	[43, 44, 50, 54]
**≥ 24 weeks follow-up**						
Anxiety (DASS-A)	5	− 0.84	− 1.40, − 0.28	0.003*	33%	[36, 38, 39, 46, 53]
Depression		SMD = − 0.36	− 0.82, 0.09	0.12	83%	[36, 38, 39, 52, 53]
DASS-D	4	− 2.39	− 4.30, − 047	0.01*	79%	[36, 38, 39, 53]
PHQ- 9	2	1.11	0.10, 2.12	0.03	0%	[39, 52]
FACIT-PAL	2	1.67	− 3.33, 6.66	0.51	0%	[34, 38]
Quality of life	6	SMD = 0.25	0.12, 0.37	< 0.0001*	22%	[36, 41, 50–52, 54]
FACT	4	3.89	0.75, 7.04	0.002*	47%	[36, 41, 52, 53]
EORTC QLQ-C30	2	5.60	− 1.10, 12.31	0.10	0%	[50, 54]

*DASS* depression, anxiety, stress scale, *PHQ- 9* Patient Health Questionnaire- 9, *FACIT-PAL* Functional Assessment of Chronic Illness Therapy-Palliative Care, *FACT-G* Functional Assessment of Cancer Therapy – General; *EORTC QLQ-C30* the European Organization for Research and Treatment of Cancer Quality of Life Questionnaire-C30; *SF- 36* Short Form Health Survey

^*^Statistically significant

^**^Unique studies refer to the number of studies analyzed, which may include multiple studies derived from the same original study but with different measurements or assessments of both short-term and long-term effects

### Short and long-term effect of EPC for family caregiver’s outcome

The meta-analysis only assessed the effects of EPC on the QoL of family caregivers. In the short and long term, neither the physical nor mental components of QoL and EPC statistically differed significantly (*p* ≥ 0.05) (See Table 5 and the forest plot in Additional File 3).

**Table 5:** Sub-group analysis of effect EPC to family caregiver’s quality of life, according measurement follow-up

Outcome	Number of studies	MD	95%CI	*p*	*I*^*2*^	Reference
< 24 weeks follow-up						
SF- 36 Physic	3	1.62	− 0.18, 3.41	0.08	0%	[22, 35, 40]
SF- 36 Mental	3	0.47	− 1.54, 2.47	0.46	0%	[22, 35, 40]
≥ 24 weeks follow-up						
SF- 36 Physic	2	− 0.01	− 1.81, 1.80	0.99	11%	[35, 40]
SF- 36 Mental	2	0.61	− 1.84, 3.07	0.62	0%	[35, 40]

*SF- 36* Short Form Health Survey

## Discussion

The present meta-analysis evaluated the effect of EPC on psychological, functional, and QoL outcomes in cancer patients and their family caregivers. The findings suggest that EPC has a significant impact on reducing anxiety, depression measured by DASS-D, and QoL among cancer patients, particularly in the long term. Improvements in functional status have not been consistently observed across studies, hence the EPC may primarily address emotional well-being rather than physical health​. The results were less encouraging for family caregivers. EPC did not lead to significant improvements in the physical or mental QoL of caregivers, both in the short and long term (< 24 and ≥ 24 weeks, respectively). However, patients and their family caregivers experienced satisfaction with the EPC.

The findings of this study are consistent with the results of previous meta-analyses conducted by Haun et al. (2017), Huo et al. (2022), Shih et al. (2022), and Cui et al. (2023), which evaluated the effects of EPC on advanced cancer patients [10, 24, 25, 56]. These previous meta-analyses demonstrated that EPC significantly improved the QoL of cancer patients. However, this present meta-analysis showed EPC significantly reduced anxiety. This finding contradicts with the current meta-analysis conducted by Cui et al. [56] reported that EPC positively affected QoL and reduced symptom burden but found no significant effects on anxiety.

EPC addresses the complex needs of cancer patients and their caregivers, especially in the context of cutting-edge personalized cancer care [57]. One of the unique mechanisms of EPC lies in its holistic approach, which integrates symptom management, psychosocial support, and personalized medical decision-making [57, 58]. Enhances illness understanding by cultivating prognostic awareness, enabling both patients and caregivers to cope with uncertainty in the face of highly variable outcomes [57]. The integrated care model of EPC ensures a seamless, compassionate, and supportive experience for patients, empowering them to make informed decisions while enhancing their quality of life, which is crucial in navigating the challenges associated with advanced cancer care [57]. These mechanisms distinguish EPC from other interventions by providing a comprehensive, patient-focused approach that spans the medical, emotional, and practical needs of both patients and their caregivers, contributing to better outcomes [57].

The present meta-analysis suggests that EPC significantly improves psychological wellbeing. This effect is likely due to the role of EPCs in alleviating emotional distress, particularly anxiety and depression [59, 60]. The EPC approach holistically incorporates psychological, physical, and emotional support at the beginning of the disease trajectory, thereby improving patients' sense of preparedness and well-being. Incorporating EPC into standard oncology care can potentially reduce the use of chemotherapy, blood transfusions, and referrals to intensive care units, improve symptom burden and mood, and increase life expectancy [61]. The capacity of EPC to resolve psychological concerns is likely a contributing factor to the increased satisfaction and QoL.

In the present meta-analysis, the QoL of cancer patients, as measured by the EORTC QLQ-C30, showed improvement after receiving at least 24 weeks of EPC. However, the effect size was relatively modest compared to findings from previous studies. Shih et al. [25] observed greater benefits, notably in long-term follow-up, while Haun et al. and Huo et al. both reported greater improvements in QoL [10, 24]. Although Cui et al. also reported improvements, they discovered a slightly stronger effect on QoL than in the current study [56]. In contrast, QoL measured using FACT did not show a significant improvement in this present meta-analysis. This discrepancy likely stems from differences in how these instruments define and assess QoL. The FACT focuses more on functional and emotional well-being, whereas the EORTC QLQ-C30 provides a multidimensional assessment, incorporating physical, role, cognitive, emotional, and social functioning, as well as symptom burden related to cancer and its treatment [62]. These variations may lead to different sensitivity levels in capturing changes over time, particularly in the context of EPC interventions. Luckett et al. [62] emphasized that, while there is no definitive psychometric evidence favoring one instrument over the other, key differences exist in their structure, emphasis on social domains, and overall approach to assessing QoL [62]. Given the variations in how these tools measure different aspects of QoL, future research may benefit from either incorporating multiple validated instruments to capture a broader perspective or developing standardized scoring methods to enhance comparability across studies.

In contrast, previous meta-analyses have focused primarily on patient-centered outcomes, providing limited insights into caregiver-related effects [25, 56]. This present meta-analysis evaluated the impact of EPC on family caregivers, although the effects on QoL were not statistically significant. The limited impact of EPC on family members may be attributed to the complexity and chronic nature of advanced cancer. Caregivers are responsible for daily care, medication administration, pain management, and emotional support, which impose substantial physical and psychological burdens [63]. Caregivers may still experience high emotional distress due to persistent caregiving demands, inadequate psychosocial support, or cultural expectations that place primary responsibility on family members [64]. This finding informs the targeted EPC addresses the psychological burden faced by caregivers.

The present meta-analysis primarily consists of studies conducted in Western countries, where family caregiving dynamics and cultural expectations differ from those in Asian countries. In many Asian societies, caregiving is seen as a familial duty rooted in cultural values like filial piety [65]. Filial culture and caregiver burden were found to have a negative association in the previous meta-analysis [65]. Family caregivers of cancer patients regard the provision of care as a societal and religious obligation, which is influenced by spiritual and religious values [66]. These cultural expectations might lead to different emotional experiences for caregivers. Unlike in Western contexts, where caregiving may be seen as an additional burden, caregivers in Asian cultures may perceive their role as a moral obligation, possibly reducing their feelings of psychological strain [66–69]. Consequently, the psychological burden observed in Western studies may not be as pronounced in Asian contexts.

In the present meta-analysis, no substantial enhancements in functional status were observed, which is consistent with the results of Gautama et al. [13], who also did not report any substantial changes in physical function. Although Shih et al.found that symptom intensity increases over time, this effect was not observed in the present meta-analysis [25]. The absence of functional status enhancement may be attributed to the fact that the physical treatments administered to the patients in both the EPC and usual care groups were similar.

Despite this present-meta-analysis show benefits, EPC faces several barriers, including delayed referrals, misconceptions that it is only for end-of-life care, limited provider training, and resource constraints [70]. Lack of awareness further hinders its utilization [70]. To address these challenges, a public health approach is essential, ensuring that EPC is integrated into all levels of healthcare. The World Health Organization (WHO) emphasizes four key components for effective palliative care implementation: (1) policy development, (2) medication accessibility, (3) education and training, and (4) service availability. However, systemic challenges persist, such as workforce shortages and inconsistent guidelines, leading to disparities in access. Overcoming these barriers requires multifaceted interventions, including policy reforms, provider education, and community engagement [71]. Therefore, expanding EPC beyond clinical settings and integrating it into public health initiatives, palliative care can become more accessible, reducing stigma and improving availability and accessibility to palliative care.

One of the major strengths of the present meta-analysis is the inclusion of a larger number of studies and a higher pooled sample size compared to previous meta-analyses [44]. The expanded sample allows for a more comprehensive understanding of the effects of EPC on cancer patients and their caregivers. Moreover, this study uniquely attempts to explore the impact of time by examining both the short- and long-term effects of EPC on psychological, functional, and QoL outcomes. By distinguishing between these timeframes, this analysis provides deeper insights into the sustainability of EPC benefits, which has been less explored in prior meta-analysis.

This study has several limitations that must be considered. One key limitation is that the present meta-analysis focused exclusively on patients with many types of cancer, meaning that the findings may not be applicable to a single type of cancer. Furthermore, most of the included studies were conducted in Western countries, limiting the applicability of the findings to non-Western contexts such as Asian countries, where cultural factors may significantly influence caregiving experiences. Outcome measurement tools and the timing of EPC interventions could have introduced heterogeneity in the results. The use of various QoL instruments, such as FACT and EORTC QLQ-C30, may have contributed to variations in reported outcomes, as these tools assess different domains of well-being with varying sensitivity. Future research should consider employing standardized QoL measures or harmonized scoring methods to enhance comparability across studies. The timing of EPC initiation varied across studies, potentially influencing observed effects. Additionally, A meta-regression was not performed due to limited study characteristics and insufficient data for each outcome, which may limit the ability to explore sources of heterogeneity in greater depth.

Our search strategy prioritized sensitivity over specificity by not restricting keywords to primary outcomes, which may have led to the inclusion of studies with limited relevance to our analysis. While we differentiated between short-term and long-term effects of EPC, the available data were insufficient to conduct a direct comparison of specific outcome metrics over time. While most studies had a low risk of bias, several were rated as having high risk, particularly due to missing outcome data and concerns about selective reporting. These biases may affect the reliability of effect estimates and the overall interpretation of findings, hence, future research should minimize these biases through improving data completeness and ensuring transparent reporting of outcomes. Variability in reporting across studies limited our ability to assess the sustainability of EPC interventions, highlighting a need for future research with standardized follow-up measures.Moreover, caregiver outcomes were underrepresented in the included studies, limiting the ability to draw strong conclusions.

## Conclusion

The present meta-analysis shows that EPC reduces the psychological burden in cancer patients over the long term and improves their QoL and care satisfaction experience. The impact of EPC on patients' functional status is inconsistent, suggesting that its main advantage may be improving emotional well-being rather than physical health. EPC did not significantly improve the QoL of family caregivers. EPC should be more broadly introduced into cancer care earlier to address patient psychological issues. Future research should focus on assessing the effectiveness of EPC within the context of Asian cultures, particularly considering how family structure, social support networks, and cultural factors might influence the outcomes of EPC.

## Supplementary Information

Supplementary Material 1.

Supplementary Material 2.

Supplementary Material 3.

## Data Availability

No datasets were generated or analysed during the current study.
