# Supplementary material for: The benefits of early palliative care on psychological well-being, functional status, and health-related quality of life among cancer patients and their caregivers: a systematic review and meta-analysis
Source: BMC Palliat Care. 2025 Apr 28;24:120. doi: 10.1186/s12904-025-01737-y (PMC12036283; doi:10.1186/s12904-025-01737-y)
Supplement: Supplementary file 1 — Supplementary Material 1. [file 12904_2025_1737_MOESM1_ESM.docx]

**Additional file 1.** Searching strategy

| Database | Keyword | Retrieved | Date |
| --- | --- | --- | --- |
| PubMed | (("Palliative Care" OR "Supportive Care") AND (“Early palliative” OR "Early Intervention" OR "Early Stage") AND ("Cancer Patients" OR "Oncology Patients") AND ("Caregivers" OR "Family Caregivers")) | 38 | January 31, 2024 |
| Scopus | "Palliative Care" OR "Supportive Care" OR "Early palliative" OR "Early Intervention" OR "Early Stage" AND "Cancer Patient*" OR "Oncology Patient*" AND caregiver* OR "Family Caregiver*" | 1688 | January 31, 2024 |
| EBSCOhost | (("Palliative Care" OR "Supportive Care") AND (“Early palliative” OR "Early Intervention" OR "Early Stage") AND ("Cancer Patients" OR "Oncology Patients") AND ("Caregivers" OR "Family Caregivers")) | 39 | January 31, 2024 |
| Cochrane | "Palliative Care" OR "Supportive Care" OR "Early palliative" OR "Early Intervention" OR "Early Stage" AND "Cancer Patient*" OR "Oncology Patient*" AND caregiver* OR "Family Caregiver*" | 266 | January 31, 2024 |
