# Supplementary figures and images for: The benefits of early palliative care on psychological well-being, functional status, and health-related quality of life among cancer patients and their caregivers: a systematic review and meta-analysis
Source: BMC Palliat Care. 2025 Apr 28;24:120. doi: 10.1186/s12904-025-01737-y (PMC12036283; doi:10.1186/s12904-025-01737-y)

**Additional file 2**


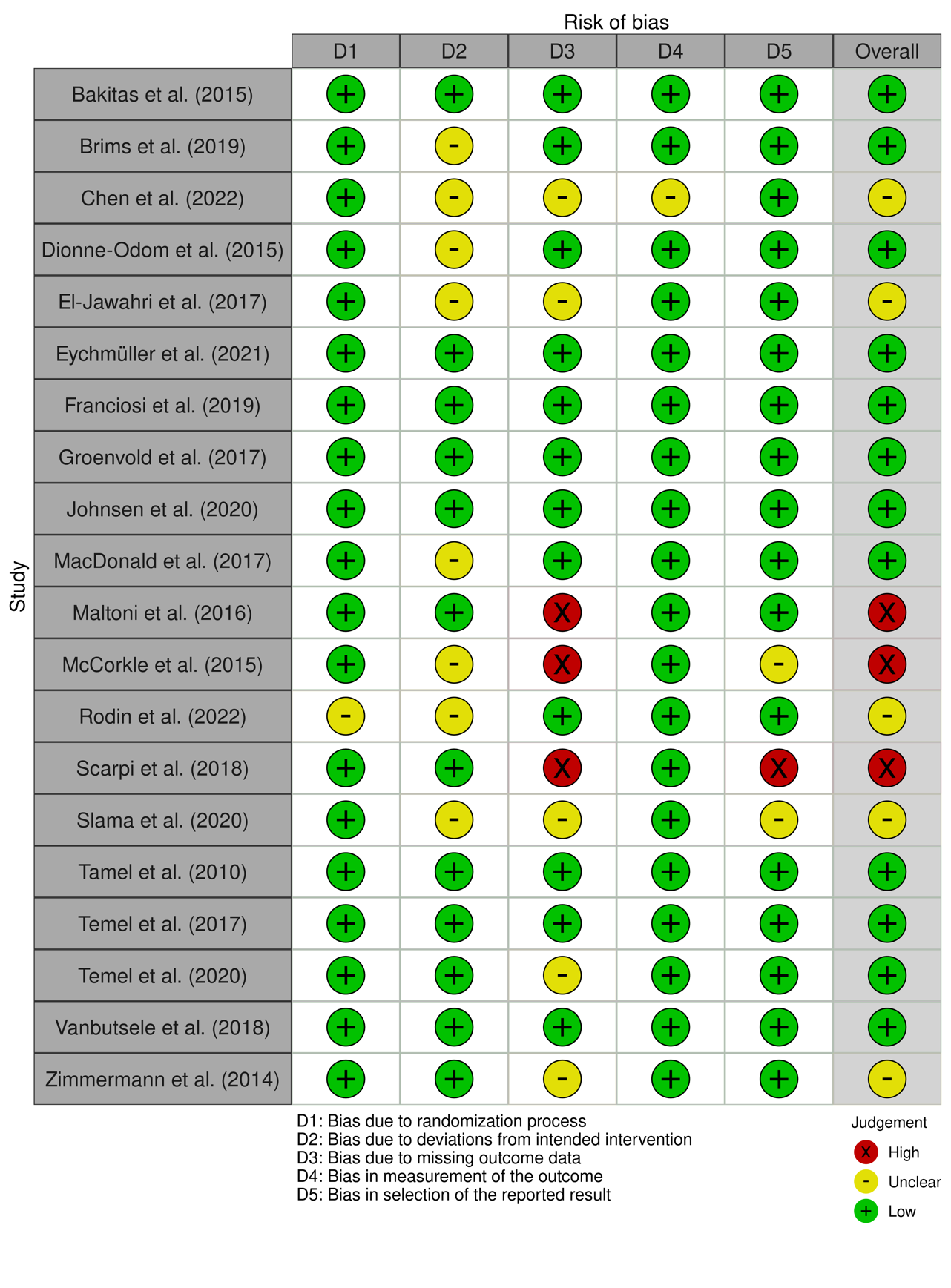


Risk of bias’s traffic light plot

Supplement: Supplementary file 2 — Supplementary Material 2. [file 12904_2025_1737_MOESM2_ESM.docx]
