# Supplementary material for: The benefits of early palliative care on psychological well-being, functional status, and health-related quality of life among cancer patients and their caregivers: a systematic review and meta-analysis
Source: BMC Palliat Care. 2025 Apr 28;24:120. doi: 10.1186/s12904-025-01737-y (PMC12036283; doi:10.1186/s12904-025-01737-y)
Supplement: Supplementary file 3 — Supplementary Material 3. [file 12904_2025_1737_MOESM3_ESM.docx]

**Additional File 3**. Forest plot


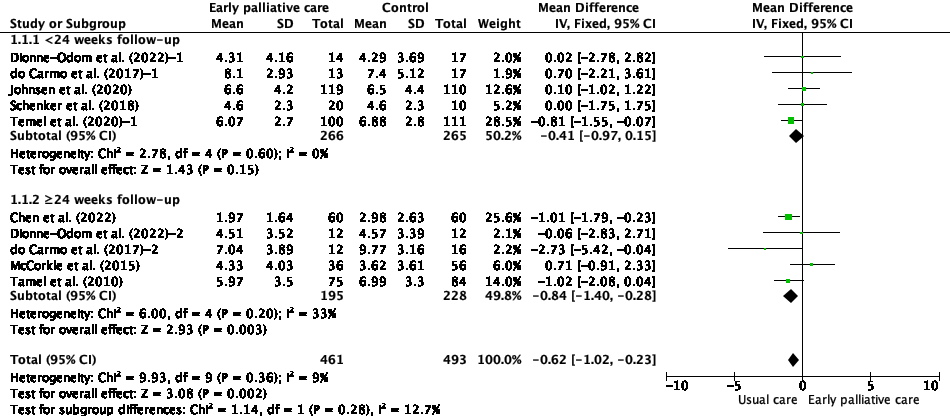


Forest plot for anxiety (DASS-A) among cancer patient


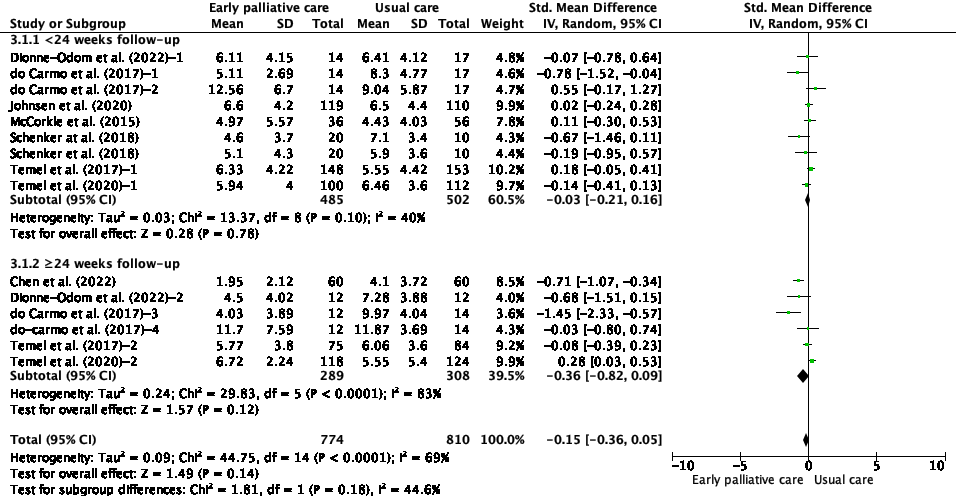


Forest plot for depression among patient cancer, using SMD


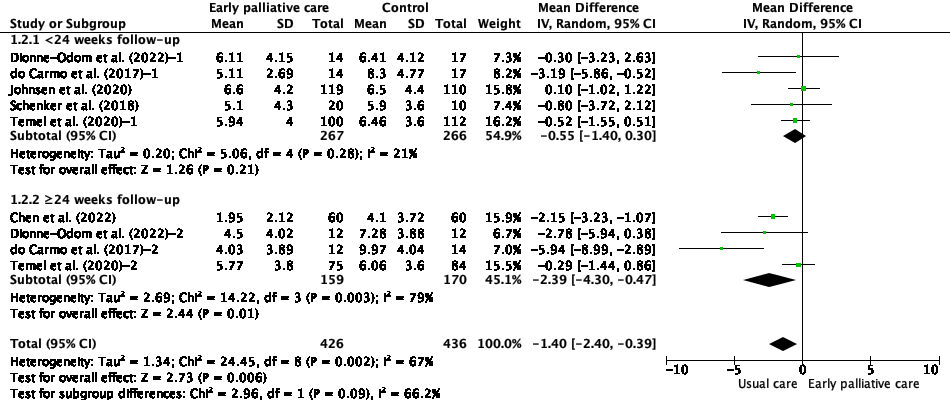


Forest plot for depression (DASS-D) among cancer patient


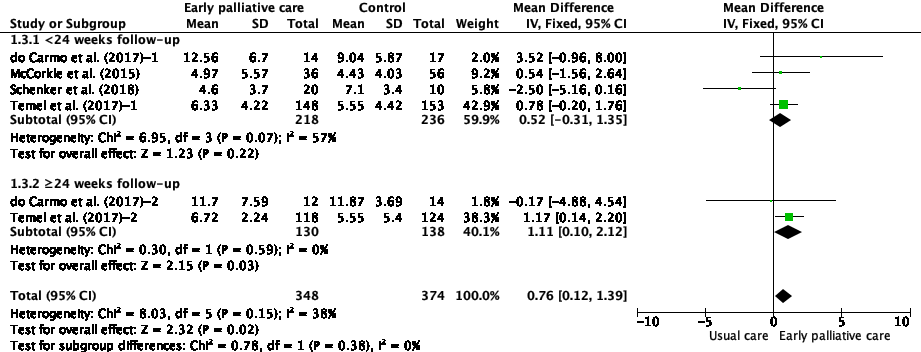


Forest plot for depression (PHQ-9) among cancer patient


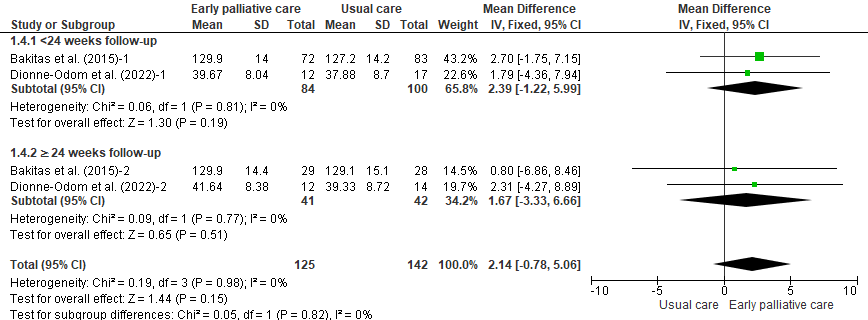


Forest plot for functional status (FACIT-PAL) among cancer patient


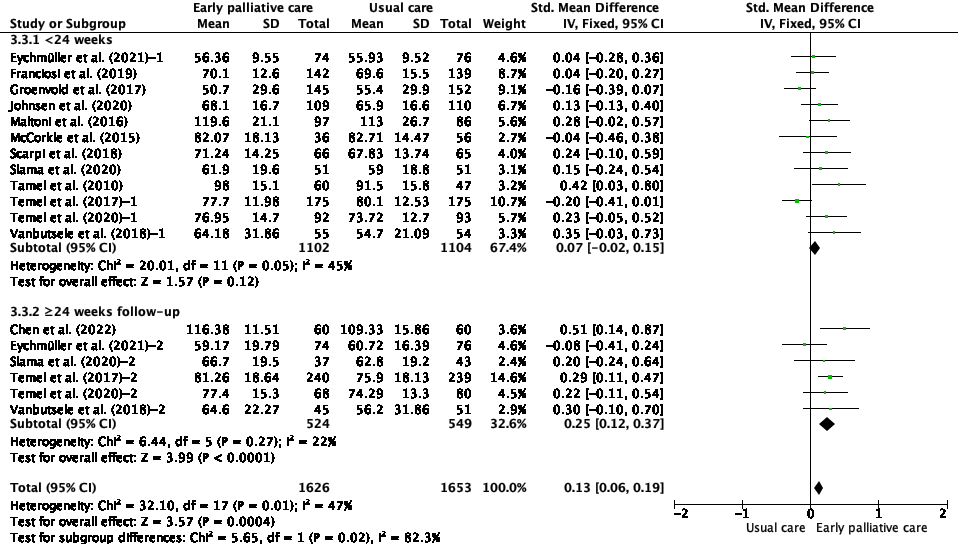


Forest plot for QoL among cancer patient, using SMD


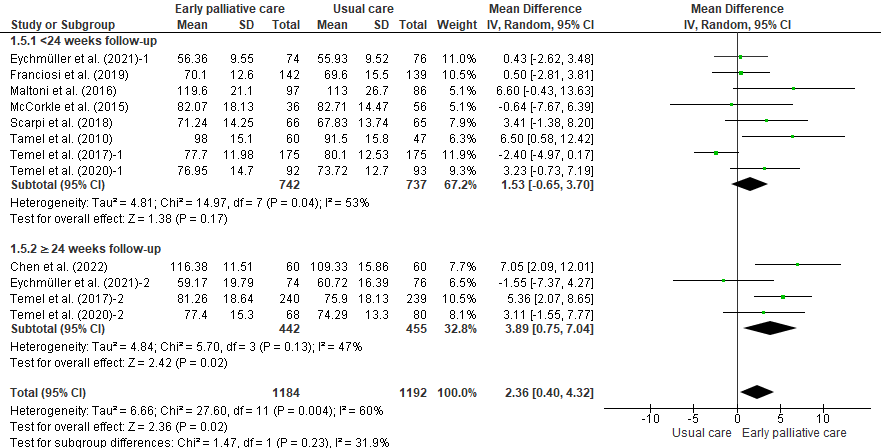


Forest plot for QoL (FACT) among cancer patient


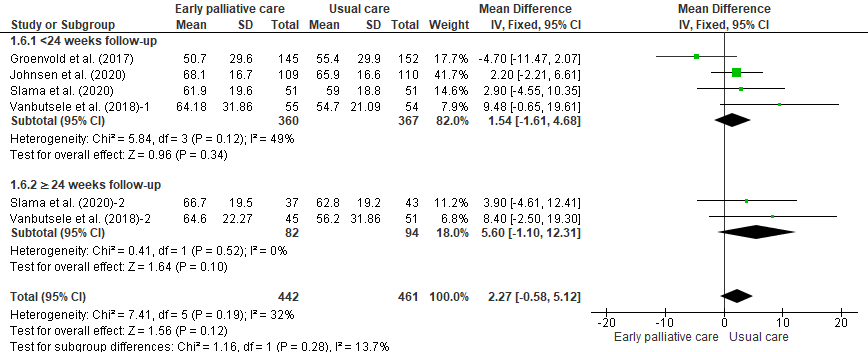


Forest plot for QoL (QLQ-C30) among cancer patient


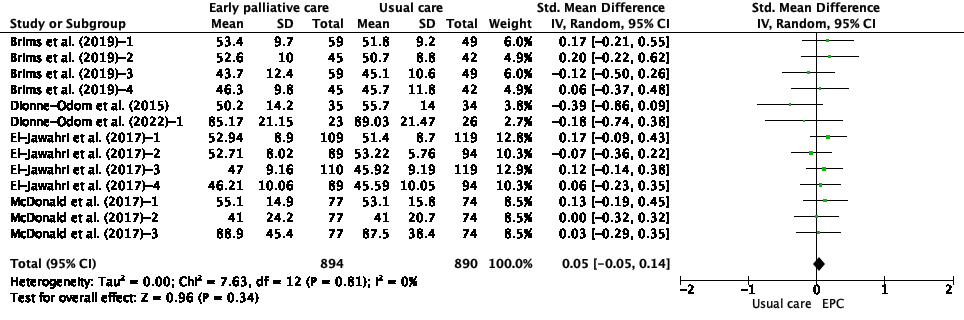


Forest plot for QoL among caregiver, using SMD


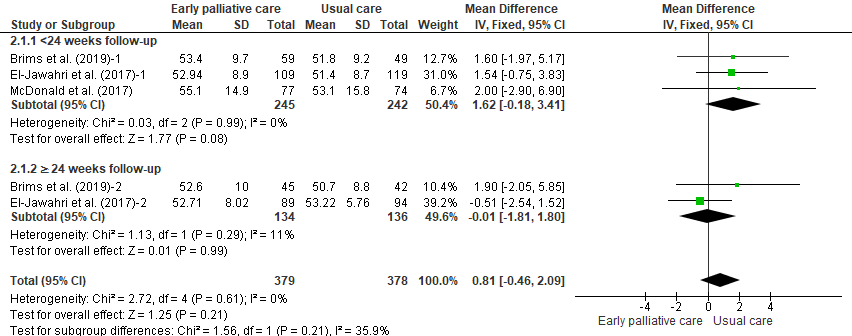


Forest plot for QoL (SF-36 physic) among family caregiver


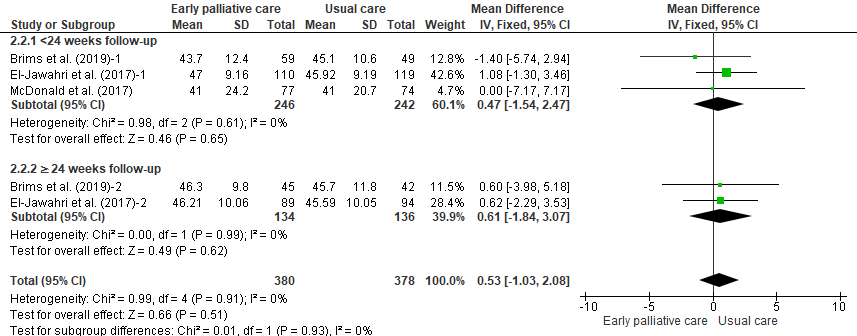


Forest plot for QoL (SF-36 mental) among family caregiver

**
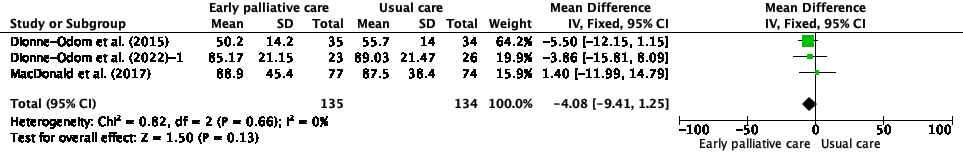
**

Forest plot for QoL (CQOL-C) among family caregiver


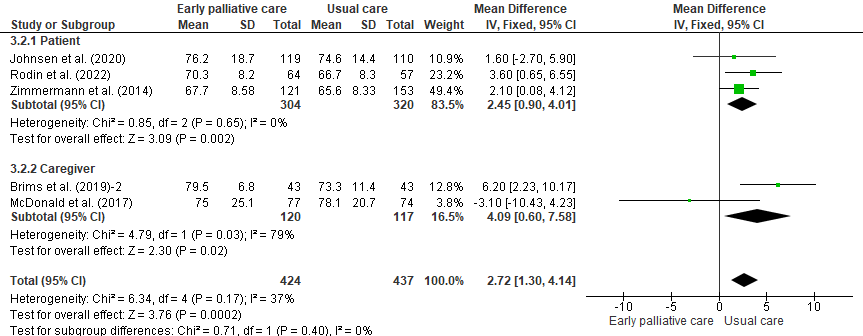


Forest plot for care satisfaction among patient and their family caregiver
